# TWENTY-FOUR-HOUR MOVEMENT AND BLOOD PRESSURE: SIX PART COMPOSITIONAL DATA ANALYSIS STUDY FROM THE PROPASS CONSORTIUM

**DOI:** 10.1093/geroni/igae098.0244

**Published:** 2024-12-31

**Authors:** Joanna Blodgett, Matthew Ahmadi, Andrew Atkin, Annemarie Koster, Emmanuel Stamatakis, Mark Hamer

**Affiliations:** University College London, London, England, United Kingdom; University of Sydney, Sydney, New South Wales, Australia; University of East Anglia, Norwich, England, United Kingdom; Maastricht University, Maastricht, Limburg, Netherlands; University of Sydney, Sydney, New South Wales, Australia; University College London, London, England, United Kingdom

## Abstract

Physical activity has widely known benefits for cardiovascular health. However, the influence of movement across the entire 24-hour behavioural spectrum (e.g. sleeping, sedentarism, activity type, etc.) on blood pressure remains underexplored. This study investigated associations between free-living activities that comprise a 24-hour day and systolic and diastolic BP (SBP; DBP). Data from six cohorts within the Prospective Physical Activity, Sitting and Sleep consortium (ProPASS) consortium (n=14,761; mean age 54.2±9.6 years) were analysed. Using thigh-worn accelerometers, all activity over a 7-day period and nurse-assessed BP were collected. Individual participant meta-analyses, employing compositional data analysis, were conducted. Reallocation plots were generated to estimate BP reductions by reallocating time from one behaviour to another, highlighting the potential benefits of minimal and optimal behavioural changes.

**Results**: On average, participants spent significant proportions of their day of sleeping (7.3hrs;31%), sedentary(10.9hrs;45%), standing(3.1hrs;13%), slow walking(1.5hrs;6%), fast walking(1.1hrs;5%), and exercise-like activity(10.9min;0.01%). Increased time spent in exercise or sleep, relative to other activities, correlated with lower BP. An additional 5 minutes/day of exercise-like activity was associated with estimated reductions of -0.68mmHg (-0.15,-1.21; SBP) and -0.54 mmHg (-0.19,0.89; DBP). Clinically meaningful improvements in SBP and DBP (2 & 1 mmHg) were estimated after reallocating 20-27 minutes and 10-15 minutes, respectively, from other activities to exercise.

**Conclusion**: This study highlight the significance of exercise in BP management, even in small doses within everyday life. It suggests that minor adjustments to daily routines, favouring exercise, could contribute to significant BP reductions in free-living contexts.

